# Supplementary figures and images for: Cross-Sectional Study of the Prevalence of Cobalamin Deficiency and Vitamin B12 Supplementation Habits among Vegetarian and Vegan Children in the Czech Republic
Source: Nutrients. 2022 Jan 26;14(3):535. doi: 10.3390/nu14030535 (PMC8838497; doi:10.3390/nu14030535)

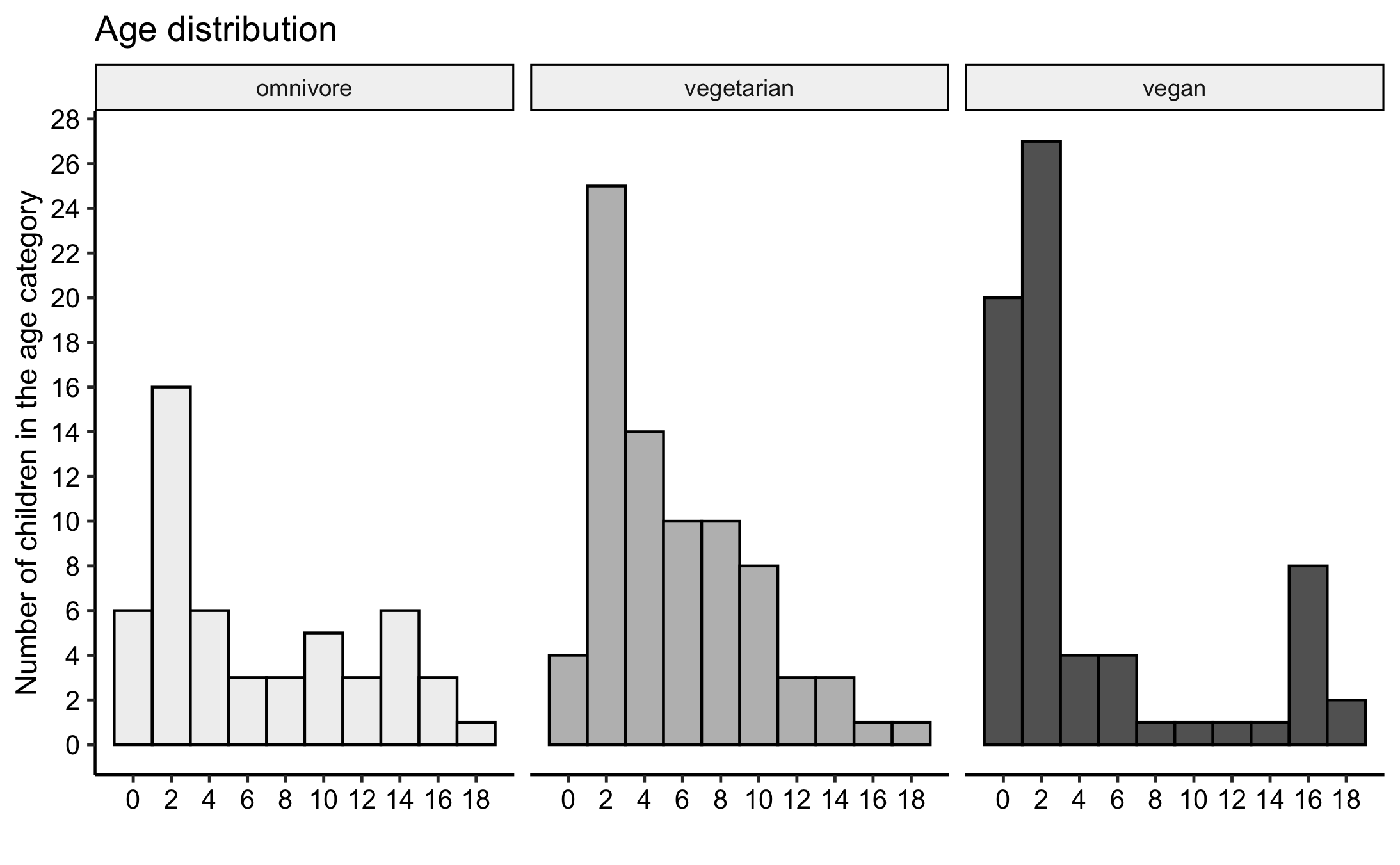

Supplement: Supplementary file 1 [file nutrients-14-00535-s001.zip › Figure S1. AgeDistribution.png]

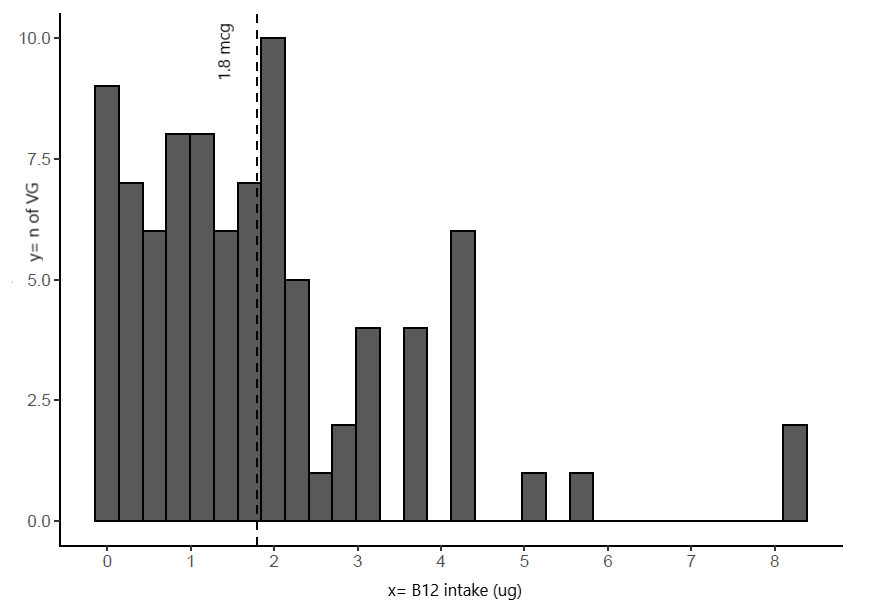

Supplement: Supplementary file 1 [file nutrients-14-00535-s001.zip › Figure S2. AvgIntakeVG.png]
